# Mindfully Green *and* Healthy: An Indirect Path from Mindfulness to Ecological Behavior

**DOI:** 10.3389/fpsyg.2017.02306

**Published:** 2018-01-18

**Authors:** Sonja M. Geiger, Siegmar Otto, Ulf Schrader

**Affiliations:** ^1^Department of Individual Differences and Psychological Assessment, Institute of Psychology and Education, Ulm University, Ulm, Germany; ^2^Department of Social Psychology, Faculty of the Natural Sciences, Otto von Guericke University Magdeburg, Magdeburg, Germany; ^3^Department of Economic Education and Sustainable Consumption, Institute for Vocational Training and Work Studies, Technische Universität Berlin, Berlin, Germany

**Keywords:** mindfulness, health behavior, ecological behavior, sustainability, sustainable consumption, mediation relationship, co-benefits

## Abstract

This paper examines the nature of the link between mindfulness and ecological behavior. Based on the notion that mindfulness incorporates heightened awareness of bodily sensations, we suggest an indirect path from mindfulness to ecological behavior that is mediated through individual health behavior, such as improved nutrition and increased exercise. This indirect path is corroborated with two online studies (*n* = 147/*n* = 239) where mindfulness, personal health behavior and ecological behavior were assessed. We conclude that increased mindful awareness of momentary experience indeed favors more healthy lifestyles, which in turn relate to increased ecological behavior beyond personal health benefits. The findings support an agreeableness of personal and planetary health behavior and open up a path for environmental educational interventions based on mindfulness practices and personal health gains.

## Introduction: mindfulness and ecological behavior

There has been a growing research interest in mindfulness over the last decade, spurred by the manifold positive effects of mindfulness evidenced in diverse fields as psychological and physical health (Grossman et al., 2004), cognitive functioning (e.g., Malinowski, 2013), and affective states (e.g., Killingsworth and Gilbert, 2010). That mindfulness might also be positively related to ecological behavior has been suggested in various recent studies (e.g., Brown and Kasser, 2005; Amel et al., 2009; Barbaro and Pickett, 2015; Panno et al., 2017). Since the concepts of mindfulness and ecological behavior applied in these studies vary considerably, we start by defining both concepts.

Based on the works of Kabat-Zinn (1991) and Bishop et al. (2004) we define mindfulness as *the awareness of momentary experiences with a non-identified, unbiased, open, and accepting attitude*. The definition comprises the two recurring aspects of mindfulness described in the literature as *awareness* and *acceptance* (Rau and Williams, 2016). The first aspect is expressed in heightened *awareness* for internal and external stimuli and sensations and the capacity to act consciously without being distracted (*acting with awareness*, e.g., Baer et al., 2008). The second aspect refers to the quality of the attitude in which people experience this awareness, namely *accepting* and *non-identified*, expressed in the ability to impartially allow whatever sensation or thought arises in a given moment without identifying with it, evaluating it or reacting to it (see also Bishop et al., 2004). Authors who emphasize the root in Buddhist ethics, regard an inner stance of equanimity, openness, and friendliness as an integral aspect of mindfulness (Shapiro and Carlson, 2009; Grossman, 2015). Despite this multifaceted nature of the construct, various authors give factor-analytical evidence for the existence of a general mindfulness factor (Walach et al., 2006; Baer et al., 2008; Bergomi et al., 2014). The general characteristic of being mindful as a state accompanying and following meditation practice, is also considered an individual difference that can be cultivated via meditation practice over time (Kabat-Zinn, 2003; Rau and Williams, 2016).

Furthermore, we define ecological behavior in line with environmental psychology approaches as *behaviors that protect/avoid harm to the environment* and span all areas of life such as nutrition, mobility and transportation, energy and water consumption, waste avoidance, and consumerism (Kaiser, 1998; Gatersleben et al., 2002; Brown and Kasser, 2005; Steg and Vlek, 2009; Geiger et al., 2017). In terms of Stern's theory toward environmentally significant behavior (Stern, 2000) we focus on individual, private sphere conservation behaviors that were successfully related to dispositional mindfulness in former studies.

That mindfulness could be related to sustainable individual behavioral choices was first suggested in the field of consumer research, where mindfulness is believed to play the role of an antagonist to impulsive, automated acquisition habits that amount to unsustainable consumerism (Kottler, 1999; Rosenberg, 2004). In a recent systematic literature review, Fischer et al. (2017) identified three more potential ways in which mindfulness could exert a positive effect on ecological behaviors in general, namely the closure of the so-called attitude-behavior gap, re-orientation toward non-materialistic, simple lifestyles, and cultivation of pro-social, compassionate behaviors.

We will outline the latter two potential ways, relevant to our own approach. Ericson et al. (2014) summarize existing evidence on how increased mindfulness may lead people to re-think their values and increase subjective well-being independently from material consumption. In various studies mindfulness was related to increased subjective wellbeing and life satisfaction (Brown et al., 2009) and the endorsement of intrinsic, non-materialistic values and ecological conservation behaviors (e.g., Brown and Kasser, 2005).

Unveiling a further potential of mindfulness to increase ecological behavior, various studies have shown that mindfulness meditation increases pro-social behavior (Leiberg et al., 2011) and compassion for other people (Lim et al., 2015), both eventually increasing ecological behavior (Corral Verdugo et al., 2011; Pfattheicher et al., 2015; Geiger and Keller, 2017). More directly, Panno et al. (2017) showed that a positive relationship between trait mindfulness and ecological behavior was mediated through decreased social dominance orientation using a known group approach. This personality trait of questioning existing inequalities between social groups has been repeatedly shown to relate to environmentalism (Milfont et al., 2017). Barbaro and Pickett (2015) focused on biospheric instead of altruistic concerns and showed that the relationship of mindfulness on ecological behavior is mediated by their connectedness to nature (Mayer and Frantz, 2004). In an experimental study by Tang et al. (2017), biospheric and altruistic cognitive foci were compared to a selfish focus. Results indicated that mindful learning techniques are only instrumental for ecological behavior when the mind is set on the environment or other people, but could be detrimental if a selfish focus is predominant.

So far, studies that empirically tested the supposed relationship between mindfulness and ecological behavior used different mediators, widely varying operationalizations of both concepts and report partially inconsistent correlations at the facet level of mindfulness (see Table 1). Most of these studies focused on *self-transcendental* values according to Schwartz's (1992) value model, such as pro-social, altruist, or biospheric concerns. In contrast, we suggest a mediation model based on *self-enhancement*, namely the improvement of personal health. Thus, we challenge the notion that an egoistic orientation is necessarily detrimental to environmental outcomes.

**Table 1:** Comparative first order correlations found between different facets of mindfulness and different ecological behavior measures.

	**Amel et al. (2009)^a^**	**Brown and Kasser (2005) study 2**	**Panno et al. (2017) study 1**	**Barbaro and Pickett (2015) study 1**	**Barbaro and Pickett (2015) study 2**	**Current paper study 1**	**Current paper study 2**
Ecological behavior (item n):	Green identity (1)	EFQ (12)/ERB (54)	ERB (17)	PEB (17)	PEB (17)	GEB (44)	GEB (50)
Mindfulness (item n):	FFMQ (8+8)	MAAS (15)	MAAS (15)	FFMQ (39)	FFMQ (39)	KIMS (20)	CHIME (37)
Mediator/Co-Variate	None	Intrinsic values	Social dominance orientation	Connectedness to nature	Connectedness to nature	Health behaviors	Health behaviors
1. Non-judging/Accepting				−0.06	0.04	0.07	0.02
2. Acting with awareness	0.37^**^	0.20^*^/0.13^**^	0.20^**^	0.02	0.15^*^	0.00	0.04
3. Awareness/Observing	0.10			0.28^***^	0.37^***^	0.18^*^	0.35^**^
4. Non-reactivity decentering				0.28^***^	0.30^***^		0.17^*^
5. Describing				0.11^*^	0.17^*^	0.20^*^	
6. Openness							0.06
7. Insight							0.17^*^
8. Relativity							0.25^**^

* *p < 0.05*,

** *p < 0.01*,

*** *p < 0.001*.

a *The coefficients of this study are standardized beta weights, as no correlations were reported, gray cells indicate facets not measured in a given study*.

*Ecological Behavior scales: EFQ, Ecological Footprint Questionnaire (Dholakia and Wackernagel, 1999); ERB, Environmentally responsible behavior; PEB, Pro- environmental behavior scale amended to students, Green identity, self-rating scale of “greenness”; GEB, Rasch based scale for General Ecological behavior (Kaiser and Wilson, 2004)*.

*Mindfulness scales: MAAS (Mindful Attention Awareness Scale, Brown and Ryan, 2003), FFMQ, Five Facet Mindfulness Questionnaire (Baer et al., 2008); KIMS, Kentucky Inventory of mindfulness (Baer et al., 2004); CHIME, Comprehensive Inventory of Mindfulness Experiences (Bergomi et al., 2014)*.

*Significant results are displayed in bold*.

## The mediation model: an *indirect* path from mindfulness to ecological behavior via health behavior

**Figure 1:**
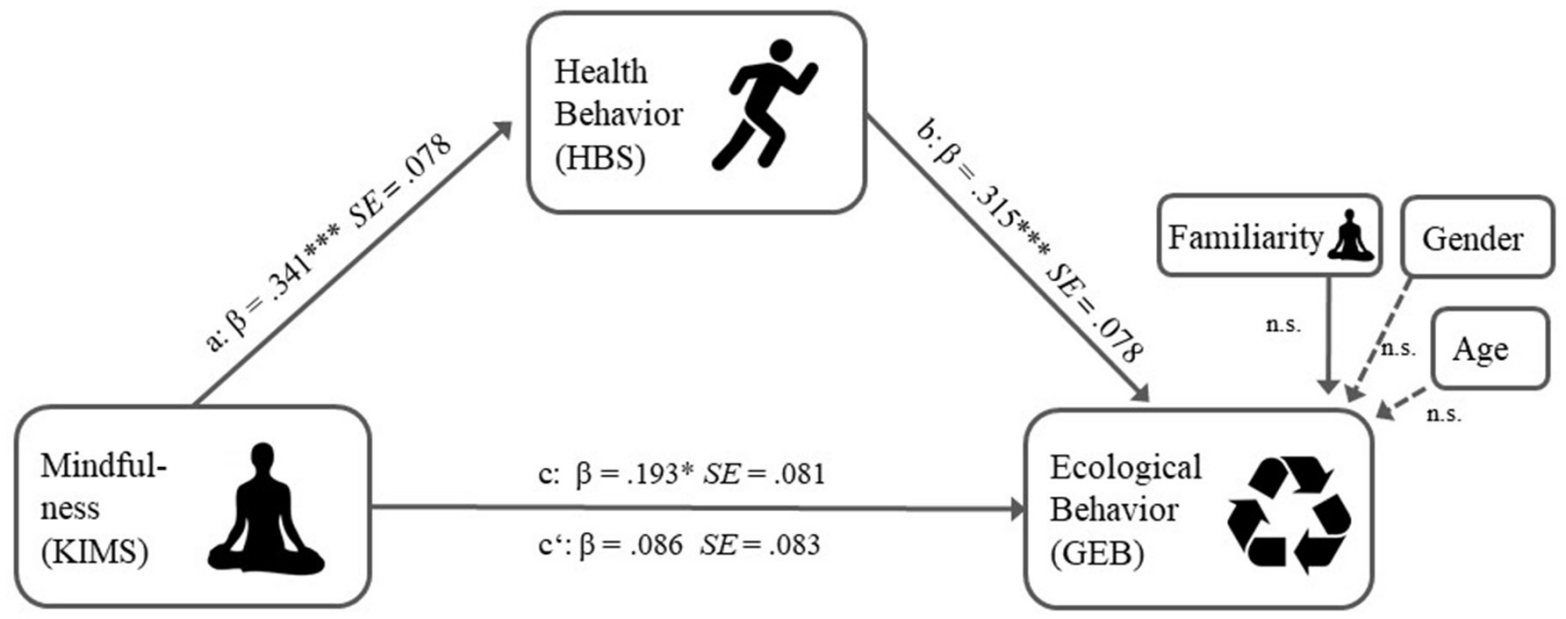
Mediation model from study 1.

That mindfulness practice entails health and environmental co-benefits at the same time was suggested in a recent conceptual paper by Barrett et al. (2016). Unlike Barrett et al. who explicitly bank on positive side-effects of mindfulness-induced-behaviors (as e.g., vegetarian nutrition) on health *and* the environment, we propose a mediation model beyond simple co-benefits, in which increased mindfulness ignites increased health behavior, which in turn is positively related to general ecological behavior even without health benefits (see Figure 1). This supposition is backed up by a substantial body of empirical research for path a (from mindfulness to health behavior). For path b (from health behavior to ecological behavior) there are various conceptual considerations and recent evidence suggesting such a relation.

### Path a

Various studies evidence the positive effects of mindfulness on psychological *and* physical health (Grossman et al., 2004; Brown et al., 2007). Based on this effect, mindfulness trainings were initially designed to treat chronic pain patients (Kabat-Zinn, 1982) and its applications were later expanded to alleviate symptoms of a wide range of (severe) illnesses (Grossman et al., 2004). Positive mental health effects comprise increased well-being, emotional stability or reduced rumination and stress (Brown and Ryan, 2003; Eberth and Sedlmeier, 2012). Especially relevant for our mediation model are the numerous studies that show how dispositional mindfulness is positively related to a variety of health behaviors (Roberts and Danoff-Burg, 2010). Furthermore, mindfulness training can support people to build new health routines for a range of behaviors such as exercising, dieting, and healthy eating (Gilbert and Waltz, 2010; Beshara et al., 2013; Salmoirago-Blotcher et al., 2013; Bahl et al., 2016). Mindfulness practice has also been reported to play a positive role in risk related health choices such as substance abuse and smoking (Black et al., 2012; Karyadi et al., 2014). Overall, a general positive effect of mindfulness practice on the intentions to take care of one's body *and* the ability to implement these intentions has been evidenced (Chatzisarantis and Hagger, 2007; Dutton, 2008; Ruffault et al., 2016). We see one reason for the observed effects in the nature of prominent mindfulness practices: they are focused on inner bodily processes and sensations, as observing the breath or body parts for prolonged periods of time. Moreover, mindfulness is partially *defined* as a heightened awareness for inner, bodily sensations in the present moment (Kabat-Zinn, 1991; Bishop et al., 2004). This heightened awareness can serve as a motivational basis for the implementation of healthier lifestyles, as expressed in healthier eating or increased exercise.

### Path b

The fact that a lot of health behaviors have co-benefits for the environment and vice versa is seen as one reason for an apparent relationship between health and ecological behavior and was exploited by Barrett et al. (2016) for their climate-activism mindfulness training. Biking to work, eating more organic and non-processed food, or moderate home temperature settings are all examples for behaviors that are beneficial to individual health while also protecting natural resources and could be framed as either (Bopp et al., 2011). Beyond direct co-benefits, the idea of a general interdependence between human and planetary health has been the focus of conceptual work by Nisbet and Gick (2008). The authors stress the dependency of human health on an intact environment and the need for environmental protection as a precondition for leading a healthy life. This notion was empirically tested for women's health behaviors, as being especially vulnerable to environmental depletion. Kim (2017) showed, that woman's individual health behavior in a South Korean sample was substantially correlated with sustainable consumption behaviors, and to a lesser degree with vicarious, social-sustainable behavior, and chemical exposure prevention (the latter being a case of behavior with co-benefits in both areas).

Other authors (Kjell, 2011; Corral Verdugo, 2012) focus on the positive consequences of sustainable behavior, such as increased (psychological) well-being, that helps spurring a positive feedback loop and asserts a positive relation between ecological behavior and health. These general thoughts are supported by the fact that throughout different nations and cultures, nature settings are appreciated for recreational purposes including health benefits (Maller et al., 2006) and well-being (Carrus et al., 2017) which in turn, is also a strong motivation for ecological behavior. Hartig et al. (2001) found, for example, that fascination with natural scenery and perceived usefulness for intended activities (including exercising) predicted general ecological behavior measured with the same instrument than in the current study. If the general line of argumentation is true, we should be able to observe a positive relation between health and ecological behavior even when direct co-benefits are absent (e.g., recycling, conserving energy and water, or buying reusable household products).

**Figure 2:**
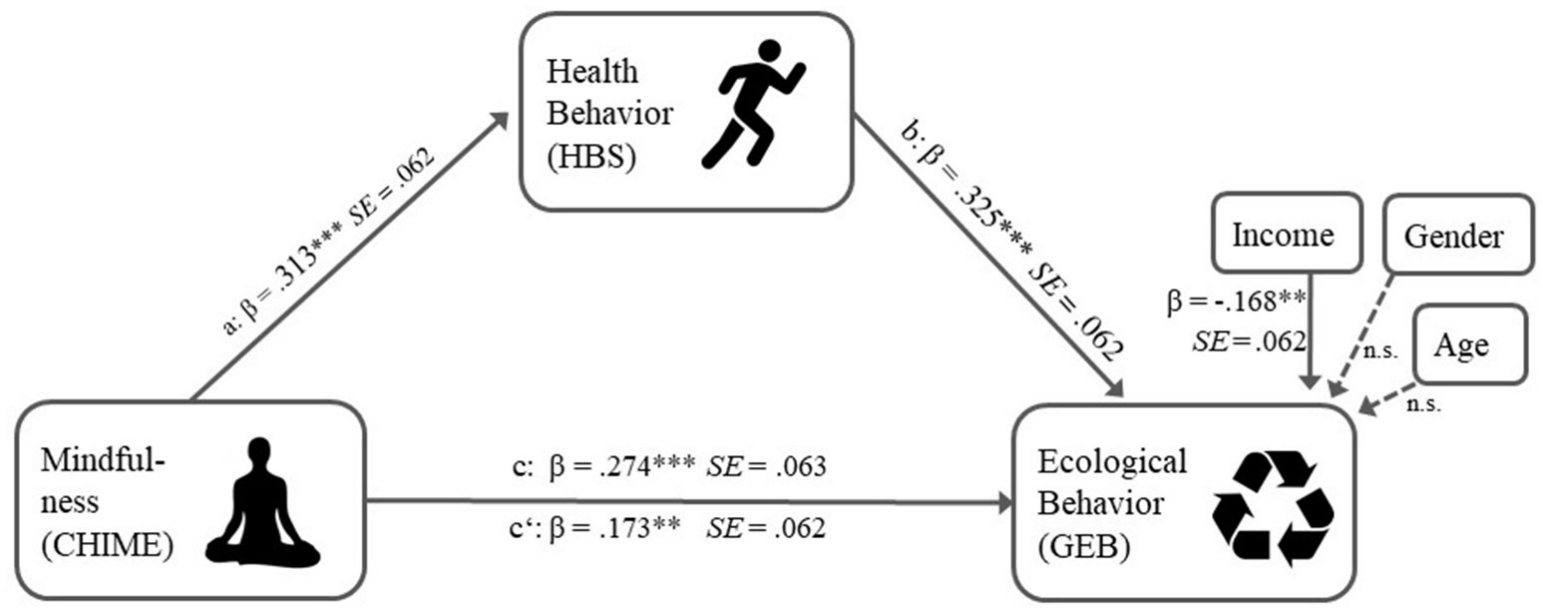
Mediation model from study 2.

In contrast to the existing conceptualizations of the relation between mindfulness and ecological behavior, our proposed mediation model is based on a self-interest to enhance one's own health and well-being. Thus, we call it—non-judgmentally—an “egocentric” path that goes beyond simple co-benefits as it relates to ecological behavior *without* explicit health benefits. Behind this background, the main aim of this study is to test, if the relation between mindfulness and ecological behavior is mediated by health behavior. This aim is pursued in two independent studies (see Figures 1, 2).

## Method of study 1 and 2

As the two studies only differed in terms of the scale used to measure mindfulness and incorporated covariates, they are reported together. The second study was included to add empirical evidence to the passionate debate on the true nature of mindfulness and its adequate assessment (e.g., Brown et al., 2011; Grossman, 2011). That mindfulness is not only a heightened capacity for paying attention, but additionally characterized by an ethical mind-set, is not fully accounted for in the KIMS scale used in study 1 (Baer et al., 2004), unlike the CHIME scale used in study 2 (Bergomi et al., 2014). This scale, validated in German, was published only after the design of study 1 in 2014, why we run a replication study with this improved measurement of our core construct.

### Procedure

Two convenience samples were recruited in social networks within a German university context, between December 2014 and February 2015 (study 1) and December 2015 and March 2016 (study 2). No reimbursement was paid. Respondents completed an online-questionnaire that assessed mindfulness, ecological behavior, health behavior, socio demographics, and three control questions about previous experience with mindfulness practices (only study 1). If more than 6 answers were missing (marking a gap in the frequency distribution of missing data), data was excluded from analysis. The studies were conducted according the ethical guidelines for online studies of the German Society for Online Research (DGOF, 2007). Consent of each participant was requested in digital form on the first page of the survey and anonymity of participants was guaranteed.

### Participants

#### Study 1

Of the initial 179 voluntary participants who started the questionnaire, 147 completed it, of which 59.2% were female. Mean age was 28.5 years (ranging from 17 to 60), with an overrepresentation of students (61.9%, compared to other work/professional situations: 23.8% employed, 5.4% self-employed and 3.4% currently jobless). The sample is highly educated (40.8% with a university entrance diploma and another 46.3% with a completed university or tertiary degree). Regarding the three control questions on previous experience with mindfulness practice, more than half of the participants, 52.4%, had heard about the concept of mindfulness before, whereas only 12.2% had participated in a mindfulness training and 2% (3 persons) identified themselves as Buddhist.

#### Study 2

Of the initial convenience sample of 295 participants, *n* = 239 completed the questionnaire, of which 67.8% were female. Mean age was 31.8 years (ranging from 18 to 79), with a lower proportion of students (35.1, vs. 44.8% employed, 5.0% self-employed and 4.2% currently on job search). The sample is also highly educated (79.9% with a university entrance diploma and 69.7% with a completed university or tertiary degree).

### Measures

#### Mindfulness

##### Study 1 (KIMS)

To assess mindfulness, a 20 item long German short version of the *K*entucky *I*nventory on *M*indfulness *S*kills (KIMS; Baer et al., 2004) by Höfling et al. (2011) was employed. The KIMS scale is aimed at people without meditation experience. The used version measures the four facets of the original scale with 5 items each: *non-judging* (e.g., “I criticize myself for having irrational or inappropriate emotions”), *acting with awareness* (e.g., “I am exclusively concentrating on what I am doing“), *observing* (e.g., “When I take a shower I stay alert to the sensation of water on my body”) and *describing* (e.g., “I am good at finding words to describe my feelings”). The German version showed a good model fit for the 4-factor structure, a reasonable convergent validity with MAAS scale and discriminant validity with the Becks Depression Inventory (Höfling et al., 2011). The 20 items were rated on a five point Likert scale from 1 (never or rarely true) to 5 (very often or always true). The mean score of all 20 items was computed after reverse coding the nine negative items along with the mean scales of the four subfacets, with higher scores indicating a more mindful person. Cronbach's Alphas for the scale and all subscales are reported in Table 2.

**Table 2:** Zero–order correlations and descriptive statistics for study 1.

**Variable**	**α**	**1.**	**2.**	**3.**	**4.**	**5.**	**6.**	***M***	**Sd**
1. KIMS	0.81							2.43	0.43
2. Describe	0.83	0.65^***^						2.67	0.67
3. Non-judging	0.82	0.68^***^	0.30^**^					2.66	0.76
4. Acting	0.75	0.50^***^	0.15^**^	0.15				1.82	0.64
5. Observe	0.76	0.64^***^	0.16^*^	0.15	0.17^*^			2.46	0.65
6. Health behavior^a^	0.67	0.34^***^	0.21^*^	0.21^**^	0.23^*^	0.21^*^	–	0.17	0.60
7. Ecological behavior^a^	0.79	0.19^*^	0.20^*^	0.07	0.00	0.18^*^	0.35^***^	−0.02	0.90

* *p < 0.05*,

** *p < 0.01*,

*** *p < 0.001*.

*N = 147*.

a *The values for HB and GEB are Rasch-based person ability scores expressed in logits and the internal consistency measure is the Rasch separation reliability correlations between the three core variables are highlighted in gray*.

*Significant results are displayed in bold*.

##### Study 2 (CHIME)

The *C*ompre*h*ensive *I*nventory of *M*indful *E*xperiences by Bergomi et al. (2014) comprises 37 items to measure seven different facets, reflecting the multifaceted nature of mindfulness. The first three, akin to the according KIMS facets, are called *acceptance* (e.g., “I see my mistakes and difficulties without judging myself”), *acting consciously* (e.g., “It is easy for me to stay focused on what I am doing”*), awareness* (e.g., “When I am sitting or lying, I perceive the sensations in my body”). Further facets are called *decentering* (e.g., “In difficult situations, I can pause for a moment without reacting immediately”)*, openness* (e.g., “I try to stay busy to keep specific thoughts or feelings from coming to my mind”), *relativity* (e.g., “In everyday life, I am aware that my view on things is subjective and does not necessarily correspond to facts”) and loving *insight* (e.g., “When I have needlessly given myself a hard time, I can see it with a bit of humor”). The CHIME showed good convergent validity with a German Version of the FFMQ (Five facets mindfulness questionnaire, Baer et al., 2008) and discriminant validity with the brief symptom inventory (BSI, for detailed information see Bergomi et al., 2014). The 37 items were rated on a six point Likert scale from 0 (never or rarely true) to 5 (very often or always true); seven items are formulated negatively and were reverse coded. Following the example of Bergomi et al. (2014), reporting good model fits for a second order general factor model with seven facets, we computed scale means for each facet and a mean score of all 37 items, with higher scores indicating a more mindful person. Cronbach's Alphas for the scale and all subscales show sufficient reliability (all above 0.7 as reported in Table 2).

#### Health behavior

To assess the health behavior of participants, we employed the German health behavior scale by Byrka and Kaiser (2013) based on the Campbell paradigm (Kaiser et al., 2010) in both studies. This scale consists of 56 health behavior items with a wide range of difficulty on a unidimensional Rash-type model. It comprises behaviors on nutrition, hygiene, stress recovery, risk prevention and physical exercise. The scale has been Rash modeled successfully and has been validated by showing its relation to physical health (e.g., body-mass-index), mental health resources (e.g. self-efficacy) and social health resources (e.g. income) (for more details on the scale's validity, see Byrka and Kaiser, 2013). In a Rasch-type model items as well as persons form a transitive order based on their difficulty. This means that some behaviors are relatively easy, that is, they are performed by most people (e.g., putting on a seat belt in the car) while difficult behaviors are performed by only few, very health dedicated individuals (e.g., eating a gluten free diet). Thus, Rasch scales allow scoring people according to “how far they reach” on a given trait, in this case health behaviors (or environmentalist dedication, see below).

In order to test our assumptions as conservatively as possible, we screened the items of the health scale for behaviors that might imply ecological co-benefits, which would artificially increase the correlation between health and ecological behaviors. In an independent rating, two of the authors identified four such items (e.g., “I ride a bike or walk to work or school”) which we subsequently omitted from the scale (for an overview of all applied and excluded items, see supplementary material, Appendix A). Of the remaining 52 items, 27 items (e.g., “I have a hobby”) were answered on a dichotomous scale (yes/no) whereas 25 items (e.g., “I go on day hikes”) were answered on a five-point Likert Scale from 1 (never) to 5 (very often or always). For all items, the option “not applicable” was available.

#### Ecological behavior

To assess the level of engagement in ecologically responsible behaviors, a German version of the general ecological behavior scale (GEB) from Kaiser (1998; Kaiser and Wilson, 2000, 2004) also based on the Campbell paradigm was used (for slight differences in versions used in the two studies, see Appendix B). The scale measures ecologically relevant behaviors from various areas, including energy conservation, mobility, waste avoidance and recycling, consumerism, and vicarious, social behaviors toward conservation (Kaiser and Wilson, 2004). The scale has been extensively validated as a unidimensional measure with a wide variety of samples (Kaiser, 1998; Kaiser et al., 1999; Kaiser and Biel, 2000; Kaiser and Wilson, 2004; and shown to predict environmental impact of individual lifestyles, Arnold et al., 2017).

Just like the items of the health scale, we screened the ecological behaviors for items with health benefits. Three of the 44 behaviors (i.e., 7 of 50 behaviors in study 2) were judged to have health co-benefits, and thus were excluded from the ecological behavior scale. Forty-one items (i.e., 43 items in study 2) entered the Rasch calibration (see Appendix B). Twenty-five of those were being answered on a five-point Likert scale (e.g., “I buy products in refillable packages”) and the 16 remaining items on a dichotomous scale (e.g., “I am a member of a car pool”). For all items, the option “not applicable” was available.

#### Socio-demographic variables

In addition to age and gender, in study 1 a sum score for increased familiarity with mindfulness was computed from the three control questions. In study 2, average monthly income was assessed additionally. These socio-demographic variables were entered as covariates (age, gender, income along with familiarity) in the mediation model.

### Data and scale reliability analysis

Following the logic of the original scale construction, we fitted a Rasch model (Bond and Fox, 2007) for the health and the ecological behavior scales with the package “eRm” (Mair and Hatzinger, 2007) in R 3.2.3. For both scales, answer options were collapsed into a uniform dichotomous answer format (“never,” “seldom,” and “occasionally” = “no/never”; and “often” and “very often/always” = “yes/always”). Kaiser and Wilson (2000) have suggested this procedure, because contrary to common expectations, more options on a Likert-scale make answers more arbitrary and less reliable. The quality of a Rasch model is judged on how well the data fit the model. Item infit Mean Square (MS) = 1 express a perfect fit, while values ranging from 0.7 to 1.3 are acceptable (Bond and Fox, 2007). We estimated the person ability score of each respondent in each of the two scales, setting items' mean difficulty to 0. These person ability scores were entered in the mediation analysis with higher values indicating a better performance.

For health behaviors, all items fitted well with infit MS, ranging from 0.88 to 1.15 (study 1) and 0.73 to 1.14 (study 2). Item no. 18 (“In cars, I wear seatbelts”—an obligatory behavior in Germany) was excluded from the analysis, because all respondents answered affirmatively. For ecological behaviors, the infit MS ranged from 0.79 to 1.24 (study 1) and 0.83 to 1.17 (study 2). Two items were answered affirmatively by all respondents (“After a picnic, I leave the place as clean as it was originally” and “I reuse my shopping bags”), and thus, did not contribute to the discrimination of persons.

To assess the internal consistency of the two Rasch scales, we computed the separation reliability, comparable to Cronbach's alpha. The internal consistency for the health scale in the two studies was acceptable with *r* = 0.67/0.74 and good for ecological behavior with *r* = 0.79/0.76. For both mindfulness scales, which were constructed according classic test theory, Cronbach's alpha indicated a good internal consistency (KIMS: α = 0.81 and CHIME: α = 0.85) and the overall scale means were used in the mediation analysis.

To test the significance of the indirect effects of the mediation model in both studies, we ran a bootstrap analysis recommended by Hayes (2009) and Preacher and Hayes (2008), using Model 4 of the PROCESS macro (Version 2.16) for SPSS (Version 23.0) described in Hayes (2012). Following the advice of Preacher and Kelley (2011), we calculated and reported two different types of effect sizes for the indirect effect, namely the completely standardized product of ab (ab_cs_) and the proportion of the indirect effect/total effect (ab/c) which allows to compare the size of the mediation effect of both studies.

## Results

### Study 1

The zero order correlations and descriptive statistics of the three study variables including the mindfulness facets are displayed in Table 2. Mindfulness is positively correlated with health behavior on all facets and to a lesser degree with ecological behavior, were only two of the facets (*describe* and *observe*) showed a small, positive relation.

Further, we found a substantial correlation between health behavior and ecological behavior, suggesting the existence of an indirect path. Figure 1 shows the standardized regression coefficients and according standard errors for all significant paths of the mediation model.

A bootstrap analysis testing the indirect effect according to Hayes (2013) using *k* = 5,000 sampling repetitions revealed a bias-corrected 95% confidence interval excluding the value 0 (point estimate = 0.097, *LLCI*_*2.5%*_ = 0.037, *ULCI*_*97.5%*_ = 0.201), confirming that health behavior mediates the relation between mindfulness and ecological behavior. The completely standardized size of the indirect effect was ab_cs_ = 0.108 or expressed as the ratio to the total effect ab/c = 55.67% (for different option on effect sizes for indirect effect see Preacher and Kelley, 2011).

Familiarity with the mindfulness concept, age and gender were entered in the mediation model as direct covariates to the dV. Results showed no effect for either variable (familiarity: β = 0.108, *p* = 0.34; age: β = −0.002, *p* = 0.81; gender: β = −0.081, *p* = 0.62). The results will be discussed together with the results of study 2.

### Study 2

The zero order correlations and descriptive statistics of the three study variables including the subfacets of mindfulness are displayed in Table 3. Health behavior was related positively with all mindfulness facets except *accepting* and *openness*. For ecological behavior a similar pattern emerged, with the only difference that *acting* consciously was also not related to ecological behavior. Health and ecological behavior, again, were substantially, positively related.

**Table 3:** Zero–order correlations and descriptive statistics for study 2.

**Variable**	**α**	**1.**	**2.**	**3.**	**4.**	**5.**	**6.**	**7.**	**8.**	**9.**	***M***	**Sd**
1. CHIME	0.85	–									3.00	0.49
2. Accepting (Non-judging)^a^	0.81	0.70^***^	–								2.37	0.99
3. Acting	0.60	0.42^***^	0.26^***^	–							2.98	0.89
4. Awareness (Observing)^a^	0.80	0.58^***^	0.11	−0.02	–						3.61	0.72
5. Decentering	0.77	0.76^***^	0.54^***^	0.26^***^	0.27^***^	–					2.64	0.85
6. Openness	0.57	0.31^***^	0.28^***^	0.21^**^	−0.10	0.15^*^	–				2.19	0.86
7. Insight	0.62	0.73^***^	0.45^***^	0.20^**^	0.36^***^	0.51^***^	0.06	–			3.33	0.75
8. Relativity	0.67	0.55^***^	0.21^**^	0.07	0.39^***^	0.316^**^	−0.08	0.43^***^	–		3.34	0.81
9. Health behavior^b^	0.74	0.31^***^	0.08	0.22^**^	0.29^***^	0.19^**^	0.06	0.26^***^	0.16^*^	–	−0.06	0.68
10. Ecological behavior^b^	0.76	0.27^***^	0.02	0.04	0.35^***^	0.17^**^	0.06	0.17^*^	0.25^***^	0.38^***^	0.35	0.80

* *p < 0.05*,

** *p < 0.01*,

*** *p < 0.001*.

*N = 239*.

a *The facet names in bracket stem from the KIMS denominating the comparable CHIME facet*.

b *The values for HB and GEB are Rasch-based person ability scores expressed in logits and the internal consistency measure is the Rasch separation reliability correlations between the three core variables are highlighted in gray*.

*Significant results are displayed in bold*.

Figure 2 shows the standardized regression coefficients and according standard errors obtained by running PROCESS with z-standardized variables for all paths.

As in study 1, we ran a bootstrap analysis with *k* = 5,000 repeated samples to test the indirect effect. The bias-corrected 95% bootstrap confidence interval excluded the value 0, indicating an indirect effect of mindfulness on ecological behavior, mediated through health behavior (point estimate = 0.166, *LLCI*_*2.5%*_ = 0.082, *ULCI*_*97.5%*_ = 0.290). The completely standardized size of the indirect effect was ab_cs_ = 0.102 or expressed as the ratio to the total effect ab/c = 37.08% (Preacher and Kelley, 2011). An extended mediation model with age, gender and income as covariates revealed a moderate negative influence of income (β = −0.165, *p* = 0.002) on ecological behavior, but neither of age (β = −0.005, *p* = 0.35) nor gender (β = −0.098, *p* = 0.46).

## Discussion

We corroborated our mediation model, proposing an indirect effect of mindfulness on ecological behavior through personal health behavior in two independent studies.

In a first instance, we found a consistent direct effect of mindfulness on ecological behavior (c: β = 0.19^*^/0.27^**^) in both studies, replicating the findings by Barbaro and Pickett (2015) on a facet level (see Table 1). The *awareness/observing* facet correlated strongest with ecological behavior, which supports the notion that being aware of one's body and external surroundings are conducive to ecological behavior. The positive relationship of the facets *insight* and *relativity* of the CHIME measure explains the stronger total effect in study 2: a certain distance to subjective experience, which is common to both facets, might free up time and energy to engage in more self-transcendental pursuits as ecological behavior. On the other hand, contrary to studies using the MAAS scale (Brown and Kasser, 2005; Panno et al., 2017) *acting with awareness* was not related to ecological behavior. A*cceptance/non-judging* also showed a null-effect, pointing toward the possibility that some aspects of cultivating mindfulness might be detrimental for ecological behavior, e.g., through increased compliance or disinterest in changing the—albeit unpleasant—status quo.

Our main finding though concerns the indirect effect of the relationship between mindfulness and ecological behavior: it was substantially mediated by health behavior. In both studies we found an indirect effect of a comparable standardized effect size (ab_cs_ = 0.108/0.102), making up 55.67/37.08% of the total effect c respectively, speaking for the general importance of health behaviors in the mindfulness-ecological behavior relation. Dispositional mindfulness related to our broad measure of health behavior, ranging from eating and hygiene habits, to exercise and risk prevention in both studies (see path a Figures 1, 2). While in the first study all mindfulness facets of the KIMS related equally to health behavior (see Table 2), in the second study, the *awareness/observing* facet of the CHIME related strongest to health behavior (see Table 3), supporting the idea that being aware of one's own body and the surrounding world might be a driver behind increased health inclinations.

That personal health behavior in turn is positively related to general ecological behavior (see path b in Figures 1, 2) extends the findings by Kim (2017) to a general population and a wider range of behaviors. Moreover, this relation does not simply reflect co-benefits, since all such items were excluded (see Appendixes A, B in Supplementary Material). That health conscious people are more likely to also conserve the environment beyond direct personal health gains is noteworthy. It means, that the realization of the interdependency of personal health and ecological, “planetary” health is empirically reflected in people's behavior (Nisbet and Gick, 2008; Corral Verdugo et al., 2011; Corral Verdugo, 2012). Former research mainly investigated self-transcendental *eco*centric or pro-social pathways to ecological behavior (e.g., through connectedness to nature, Barbaro and Pickett, 2015; intrinsic values, Brown and Kasser, 2005, reduced social dominance orientation, Panno et al., 2017). In contrast, our mediation model is based on a self-serving, *ego*centric interest to improve personal health. In this respect our findings corroborate former research indicating that egocentric self-enhancement values can also advocate conservation of nature for utilization motives (e.g., Milfont and Gouveia, 2006). It also complements recent publications about co-benefits of ecological behavior opening up new ways of promoting environmental action (Corral Verdugo, 2012; Bain et al., 2016). In our case, we provide empiric evidence of why mindfulness environmental educational approaches (Barrett et al., 2016; Stanszus et al., 2017) based on health *and* ecological co-benefits should be fruitful.

A strength of the current study is that all paths were tested with validated scales for the respective constructs, comprising a wide variety of subfacets each. The two behavioral scales encompass a great range of relevant behaviors instead of focusing on single behaviors (e.g., smoking or recycling), as often the case in health or environmental studies. That the results have been conceptually replicated with two different mindfulness scales underlines the robustness and general character of the findings.

Neither familiarity with mindfulness, nor age or gender did explain any proportion of behavioral variance. In the case of age, our young and unrepresentative sample might have prevented a weak relation found in other studies (for an overview of age effects in ecological behavior, see Wiernik et al., 2013; Otto and Kaiser, 2014). Regarding previous exposure to the mindfulness concept, a more detailed measure of actual meditation experience in terms of regularity and length of practice would be desirable in future studies. Assessing previous practice might have yielded more insightful results into the role of actual meditation practice in cultivating sustainable lifestyles than the short measure we used. In the second study, income was a negative predictor of ecological behavior, showing that higher income is usually detrimental to ecological behavior, corroborating findings of representative studies (e.g., Kleinhückelkotten et al., 2016). This shows, that ecological behavior could also be partially motivated by saving money, another self-centered motive.

A caveat all cross-sectional studies face, lays in the inconclusiveness with regards to the assumed causal direction. The mediation analysis of cross-sectional data does not *per se* warrant the interpretation of an intrapersonal, temporal process (Winer et al., 2016), in our case from mindfulness via health behavior to ecological behavior. Alternative models, e.g., where health problems make people turn simultaneously to mindfulness practices and ecological behavior is theoretically likewise conceivable. For further possible interpretations of mediation models, see Roe (2012). We base the interpretation of our data as an intrapersonal process on former intervention studies, which showed (for path a) that mindfulness practice fosters health behaviors such as balanced nutrition or increased exercise (e.g., Dutton, 2008; Salmoirago-Blotcher et al., 2013). This causal chain has yet to be established for path b in future research with, for example, intervention studies aiming at health behaviors and measuring ecological behaviors as a dependent variable.

A possible alternative to a causal intrapersonal process includes a common third factor, e.g. an inclination for a nature-related, spiritual lifestyle, which could be more prevalent in our voluntary samples with a propensity for the topic. As connectedness to nature was shown to mediate the relation between mindfulness and ecological behavior in a cross-sectional study similar to ours (Barbaro and Pickett, 2015), their effect could also be interpreted as a third-factor confound instead of a causal process. Appreciation of nature could be another such factor: it is partially fuelled by a health motive, i.e., people seeking restoration effects in nature settings (Maller et al., 2006; Carrus et al., 2017; see also Mercado-Doménech et al., 2017 for a recent discussion on motivation and evaluation in the perception of environmental stimuli) and has been proven to spur ecological behavior (Hartig et al., 2001; Otto et al., 2014). How these phenomena relate to each other and to mindfulness practice, health behavior and ecological behavior, will have to be explored in future studies. Furthermore, a recent study with children showed the positive results of exposure to nature for pro-social outcomes (Carrus et al., 2015) and environmental behavior (Otto and Pensini, 2017). Thus, another promising avenue for future research would be an intervention study combining nature-exposure and mindfulness practices, measuring the outcomes on health and environmental behavior.

## Conclusions

The presented evidence on the relation between mindfulness and ecological behavior favors a mediation model via personal health behavior and partially supports former research on a direct, modest link between the two concepts. Our findings suggest that the increased ecological behavior of mindful people is an indirect consequence of increased personal health behavior. The overall positive effect of mindfulness on ecological behavior seems to warrant considerations to include mindfulness trainings and practices into the intervention canon of environmental education. A combination of mindfulness and nature experiences might be an effective way to enhance the positive health effects while at the same time promoting ecological behavior. Generally, the results support a new narrative of the compatibility of self-serving, personal, and planetary health benefits which might be a more fruitful approach to promote ecological behavior than mainly cognitive, moral, or normative appeals.

## Author contributions

SG: Analyzed data, did literature research, concept of the paper, wrote the manuscript, expertise on mindfulness and ecological behavior. SO: Collected and Rasch-modeled the data, expertise on health and ecological behavior. US: Overall conceptualization on mindfulness and ecological behavior.

### Conflict of interest statement

The authors declare that the research was conducted in the absence of any commercial or financial relationships that could be construed as a potential conflict of interest. The reviewer AP, and handling Editor declared their shared affiliation.
