# Supplementary material for: Mindfully Green and Healthy: An Indirect Path from Mindfulness to Ecological Behavior
Source: Front Psychol. 2018 Jan 18;8:2306. doi: 10.3389/fpsyg.2017.02306 (PMC5778139; doi:10.3389/fpsyg.2017.02306)
Supplement: Supplementary file 1 [file DataSheet1.docx]

Appendix A The version of the health Scale used in both studies (Byrka & Kaiser, 2013)

| No |  |
| --- | --- |
| **5-point Likert items** | |
| 1 | I check the nutritional value of food products. |
| 2 | I eat after 9 p.m.* |
| 3 | I check the consumption date of food products. |
| 4 | I wash meat before preparing it. |
| 5 | I clean cans before opening them. |
| 6 | I wash dishes right after a meal or at least on the same day. |
| 7 | To let in fresh air, I open windows for a long period of time. |
| 8 | After using a restroom, I wash my hands. |
| 9 | I wash my hands bevor cooking or eating. |
| 10 | I meditate or practice yoga. |
| 11 | I go to bed before 10 p.m. |
| 12 | I spent time in nature. |
| 13 | I work overtime.* |
| 14 | I take my breaks at work. |
| 15 | I take time to relax. |
| 16 | I spend time with other people to socialize. |
| 17 | In the evening or during weekends, I work at home.* |
| 18 | I sleep at least 7 hours per night. |
| 19 | I take wellness offers (e.g. sauna, massage) |
| 20 | I cross streets on a red light* |
| 21 | I get drunk.* |
| 22 | With new medication, I read the package insert. |
| 23 | When it´s cold, I wear worm clothes. |
| 24 | I go on day hikes. |
| 25 | I rather take the elevator than the stairs.* |
| **Dichotomous items** | |
| 26 | I count calories. |
| 27 | I avoid sugar. |
| 28 | I avoid salty foods. |
| 29 | I eat fruits and vegetables daily. |
| 30 | My meals last at least 10 minutes. |
| 31 | I drink at least 2 litres a day. |
| 32 | I floss my teeth at least twice a week. |
| 33 | I allow pets in my kitchen.*. |
| 34 | I brush my teeth at least twice a day. |
| 35 | I wash fruits and vegetables. |
| 36 | I have a hobby. |
| 37 | I regularly examine myself for cancer. |
| 38 | I use sun screen. |
| 39 | At least twice a year, I have my teeth checked. |
| 40 | I drink more than a glass of wine or a beer a day.* |
| 41 | I smoke.* |
| 42 | In cars I wear seatbelts. |
| 43 | I wear a bike helmet. |
| 44 | I protect myself from sexually transmitted dieses (e.g. with condoms, vaccinations) |
| 45 | I possess a fitness video. |
| 46 | I keep an exercise diary. |
| 47 | I exercise at least 15 minutes per day. |
| 48 | I am a member of a sport facility / fitness club. |
| 49 | I go for a walk at least 15 minutes a day. |
| 50 | I possess sports gear. |
| 51 | I play sports regularly (swimming, football, etc.). |
| 52 | I possess a fitness computer console (e.g. Wii-Fit, Eye Toy Kinetic etc.) |
| **Excluded items due to co-benefits.** | |
|  | *I avoid fast food.* |
|  | *In the winter, I keep the heat on so that I do not have to wear a sweater.* |
|  | *I ride a bike or walk to work or school.* |
|  | *I buy products with eco-labels.* |

* reverse items

Appendix B, Version of the GEB Scale used in both studies (Kaiser & Wilson, 2004)

| No. |  |  |  |
| --- | --- | --- | --- |
| **5-point-Likert items** | | **Study 1** | **Study 2** |
| 1a | I prefer to shower rather than to take a bath. | x |  |
| 1b | I shower (rather than to take a bath). |  | x |
| 2 | I buy beverages in cans.* | x | x |
| 3 | I use an oven cleaning spray to clean my oven.* | x | x |
| 4 | I drive my car in or into the city.* | x | x |
| 5 | In the winter, I leave the windows open for longer periods of time to let in fresh air.* | x | x |
| 6 | I wash dirty clothes without prewashing. | x | x |
| 7 | If I am offered a plastic bag in a store, I take it.* | x | x |
| 8 | I buy seasonal produce. | x | x |
| 9 | I use a clothes dryer.* | x | x |
| 10 | I read about environmental issues. | x | x |
| 11 | I talk with friends about environmental pollution, climate change, and/or energy consumption. | x | x |
| 12 | For longer journeys (more than 6 hours of travel time by car), I take an airplane.* | x | x |
| 13 | I keep the engine running while waiting in front of a railroad crossing or in a traffic jam. * | x | x |
| 14 | At red traffic lights, I keep the engine running.* | x | x |
| 15 | In winter, I turn down the heat when I leave my apartment for more than 4 hours. | x | x |
| 16 | I go with my car to where I want to start my hikes.* | x | x |
| 17 | I collect and recycle used paper. | x | x |
| 18 | I bring empty bottles to a recycling bin. | x | x |
| 19 | I have pointed out unecological behavior to someone. | x | x |
| 20 | I contribute financially to environmental organizations. | x | x |
| 21a | I buy milk in returnable bottles. | x |  |
| 21b | I buy beverages and other liquids in returnable bottles. |  | x |
| 22 | I buy bleached or coloured toilet paper.* | x | x |
| 23 | I buy products in refillable packages. | x | x |
| 24 | I buy domestically grown wooden furniture. | x | x |
| 25 | I boycott companies with an unecological background. | x | x |
|  |  |  |  |
| ***Dichotomous items*** | |  |  |
| 26 | I reuse my shopping bags. | x | x |
| 27 | I use fabric softener with my laundry.* | x | x |
| 28 | I put dead batteries in the garbage.* | x | x |
| 29 | After meals, I dispose of leftovers in the toilet.* | x | x |
| 30 | I use a chemical air freshener in my bathroom.* | x | x |
| 31 | I am a member of an environmental organization. | x | x |
| 32 | I use energy efficient household devices. | x | x |
| 33 | After a picnic, I leave the place as clean as it was originally. | x | x |
| 34 | I own solar panels. | x |  |
| 35 | I have looked into the pros and cons having a private source of solar power. | x | x |
| 36 | I requested an estimate on having solar power installed. | x |  |
| 37a | I use renewable energy sources. | x |  |
| 37b | I have a contract for renewable energy with my energy provider. |  | x |
| 38 | I refrain from owning a car. | x | x |
| 39 | I am a member of a carpool. | x | x |
| 40 | I drive in such a way as to keep my fuel consumption as low as possible. | x | x |
| 41 | I own a fuel-efficient automobile (less than 7 litres per 100 kilometre). | x | x |
| 42 | When I leave the room, I turn off the light. |  | x |
| 43 | On freeways I drive at speeds under 100kph (= 62.5 mph). |  | x |
| 44 | I wait until I have a full load before doing my laundry. |  | x |
| 45 | In hotels, I have the towels changed daily. * |  | x |
|  |  |  |  |
| **Excluded items due to co-benefits.** | |  |  |
|  | *I buy organically produced food.* | x | x |
|  | *I kill insects with a chemical insecticide.* | x | x |
|  | *To reach nearby areas (around 30 kilometres; around 20 miles), I use public transportation or ride a bike.* | x | x |
|  | *I walk, ride a bicycle or take public transportation to work.* |  | x |
|  | *I am a vegetarian* |  | x |
|  | *I buy convenience food.* |  | x |
|  | *In winter, I keep my heating temperature settings so high, that I feel warm without having to wear a sweater.* |  | x |

* reverse items
